# CHIPIN: ChIP-seq inter-sample normalization based on signal invariance across transcriptionally constant genes

**DOI:** 10.1186/s12859-021-04320-3

**Published:** 2021-08-17

**Authors:** Lélia Polit, Gwenneg Kerdivel, Sebastian Gregoricchio, Michela Esposito, Christel Guillouf, Valentina Boeva

**Affiliations:** 1 Institut Cochin, Inserm U1016, CNRS UMR 8104, Paris Descartes University UMR-S1016, 75014 Paris, France; 2 INSERM UMR1170, Equipe Labellisée Ligue Nationale Contre Le Cancer, Gustave Roussy, Paris-Saclay University, 94800 Villejuif, France; 3 Department of Computer Science, Institute for Machine Learning, ETH Zurich, 8092 Zurich, Switzerland; 4 Swiss Institute for Bioinformatics (SIB), Zürich, Switzerland

**Keywords:** ChIP-seq, Open chromatin, Normalization, Density profiles, Gene expression, Algorithm, R package

## Abstract

**Background:**

Multiple studies rely on ChIP-seq experiments to assess the effect of gene modulation and drug treatments on protein binding and chromatin structure. However, most methods commonly used for the normalization of ChIP-seq binding intensity signals across conditions, *e.g.*, the normalization to the same number of reads, either assume a constant signal-to-noise ratio across conditions or base the estimates of correction factors on genomic regions with intrinsically different signals between conditions. Inaccurate normalization of ChIP-seq signal may, in turn, lead to erroneous biological conclusions.

**Results:**

We developed a new R package, CHIPIN, that allows normalizing ChIP-seq signals across different conditions/samples when spike-in information is not available, but gene expression data are at hand. Our normalization technique is based on the assumption that, on average, no differences in ChIP-seq signals should be observed in the regulatory regions of genes whose expression levels are constant across samples/conditions. In addition to normalizing ChIP-seq signals, CHIPIN provides as output a number of graphs and calculates statistics allowing the user to assess the efficiency of the normalization and qualify the specificity of the antibody used. In addition to ChIP-seq, CHIPIN can be used without restriction on open chromatin ATAC-seq or DNase hypersensitivity data. We validated the CHIPIN method on several ChIP-seq data sets and documented its superior performance in comparison to several commonly used normalization techniques.

**Conclusions:**

The CHIPIN method provides a new way for ChIP-seq signal normalization across conditions when spike-in experiments are not available. The method is implemented in a user-friendly R package available on GitHub: https://github.com/BoevaLab/CHIPIN

**Supplementary Information:**

The online version contains supplementary material available at 10.1186/s12859-021-04320-3.

## Background

In the last decade, chromatin immunoprecipitation followed by sequencing (ChIP-seq) became an important technology to study genome-wide protein-DNA interactions. In particular, application of the ChIP-seq technique allowed studying mechanisms of gene transcription including getting insights into the role of transcription factors and posttranslational modifications of histone proteins. For instance, it was demonstrated that histone modifications (acetylation, methylation, phosphorylation, ubiquitination and SUMOylation) were often involved in transcriptional regulation. Acetylation of lysine (*e*.*g*., the lysine 27 of the histone H3, H3K27ac) was linked to gene expression activation while methylation of lysine (*e*.*g*., H3K27me3 and H3K9me3) was often associated with transcriptional repression [1]. Using the ChIP-seq technology, exact position of histone modifications and transcription factors binding sites could be obtained.

ChIP-seq technology includes three main steps: (1) covalent crosslink of proteins such as histones or transcription factors to their genomic DNA substrates in living cells; (2) isolation and fragmentation to capture the protein-DNA complexes using an antibody specific for the protein of interest; (3) analysis of the immunoprecipitated DNA by high-throughput sequencing after reversal of cross links and purification. ChIP-seq technology has been often used to compare DNA binding of the protein of interest in two or multiple conditions. However, due to technical biases, comparing ChIP-seq signals in several conditions still remains a challenging issue. First, the signal comparison is impeded by variable efficiency of chromatin immunoprecipitation across samples. Second, ChIP-seq experiments are subject to variability in fragment length distributions and can exhibit differences in read counts caused by the DNA amplification bias. Together these biases can cause artefactual variations in ChIP-seq signal and lead to erroneous biological conclusions. Therefore, there is a strong need for a normalization procedure that could correct the aforementioned effects, especially when spike-in information [2], relying on the addition of chromatin and antibody (Spike-in) from a different organism and allowing for better inter-sample normalization, is not available. Indeed, today most experimental work involving ChIP-seq does not use spike-in information [2].

In most studies, the ChIP-seq signal normalization is performed using the total number of sequenced fragments per sample. The data is scaled by a constant factor, for instance using the function “*bamCompare*” provided by the “deepTools” package [3]. Unfortunately, this normalization approach does not account for differences in the efficiency of immunoprecipitation and DNA amplification biases. Other methods such as Normalization of ChIP-Seq (NCIS) by Liang and Keleş [4], which extended CisGenome [5], use for the signal normalization the control sample. Here, the genome is divided into non-overlapping bins; a normalization factor is calculated as $r = \frac{{\mathop \sum \nolimits_{i \in B} {\text{ChIP}}\_{\text{signal}}}}{{\mathop \sum \nolimits_{i \in B} {\text{Control}}\_{\text{signal}}}}$, where *B* corresponds to the set of bins that belong to the background. In CisGenome [5], the authors determine background regions as regions with total counts $t \le 1$ and bin-width $w = 100$, and the normalization factor $r$ is computed given these parameters. In NCIS, the marginal ChIP/Control ratio against total counts is used to determine the optimal value of $t$ and $w$ in a data-adaptive manner and finally define set *B*.

More sophisticated methods [6–8] allow the user to determine differentially enriched regions, while addressing normalization problem but do not provide the resulting normalized profiles. For instance, in ChIPnorm [7], the background is estimated, the local genomic bias is removed and finally the normalization problem is addressed using quantile normalization. MACS2, one of the most used ChIP-seq analysis tools [9], permits identifying differential peaks (function *bdgdiff*) but the resulting normalized profiles are not provided. A two-step non-linear normalization method based on locally weighted regression (LOESS) approach was developed by Taslim et al. [8]. To compare ChIP-seq data across multiple samples, the method models the difference using an Exponential-Normal_K_ mixture model and uses the fitted model to select regions associated with differential binding sites based on local false discovery rate. ChIPseqSpikeInFree [6] is a recent normalization method based on the computation of the slope of the cumulative read counts curve for each sample. Although powerful, these methods for differential peak calling do not provide normalized ChIP-seq density profiles as output.

Our team has recently developed a simple normalization method, LILY, based on matching the signal within strong peaks common to all conditions [10]. The normalization factor is computed as the ratio of density values in these common regions. Importantly, the LILY pipeline is based on the ChIP-seq profiles explicitly corrected for copy number variation [11] and thus can be used to compare signal between different cancer samples or between cancer and healthy tissues.

In sum, existing methods usually evaluate the normalization parameters based on common ChIP-seq peaks, or by using only the control, and do not accept additional information, *e*.*g*., gene expression, to establish a baseline. In this paper, we fill this gap by creating a user-friendly R package for ChIP-seq data inter-sample normalization. This method is based on the biological assumption that genes with constant expression across samples have, on average, similar protein binding intensities. Indeed, the correlation between gene expression and the deposition of chromatin marks or transcription factor binding has been documented by a number of studies [12–15] (see also Additional file 1: Figure S1).

## Implementation

**Fig. 1:**
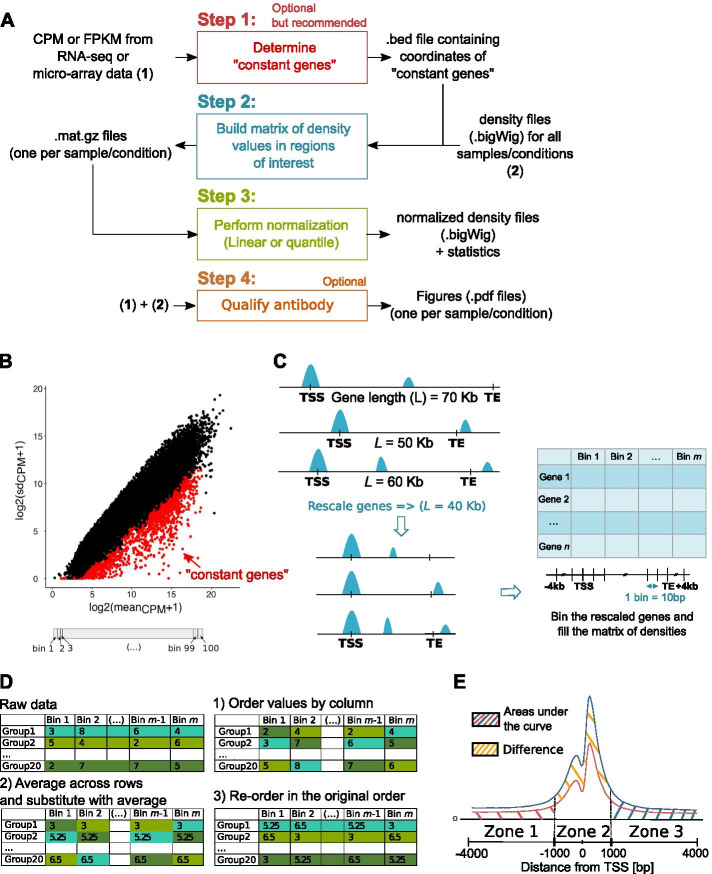
Outline of the CHIPIN package. **A** Four step approach implemented in CHIPIN. **B** Definition of “constant genes” (red) used as a baseline for the ChIP-seq signal normalization: “constant genes” are 10% (default) of genes with the lowest standard deviation of read counts selected from different ranges of gene expression (100 bins). **C** Gene body regions of “constant genes” and surrounding flanking regions (± *n* Kb, *n* = 4 Kb by default) are rescaled to 40 Kb (default) and segmented into bins (default bin length: 10 bp). TSS: transcription start site; TE: transcription end site. **D** Main steps of the quantile normalization. In CHIPIN the quantile normalization is applied separately to subsets of genes within a certain range of ChIP-seq intensity signal (*k* groups, *k* = *20* by default). **E** Difference in areas under the density curves is used for the evaluation of the success of the normalization process

We propose a normalization method called ChIPIN for ChIP-seq Inter-sample Normalization of fragment density signals across multiple conditions. Our method is based on the following assumption: on average, no difference in true ChIP-seq signal should be observed in the regulatory regions of genes whose expression is constant across samples/conditions. Our algorithm comprises three main steps (Fig. 1A). An additional, 4th step allows the user to verify the specificity of the antibody used. The tool is implemented as a user-friendly R package available online on GitHub (https://github.com/BoevaLab/CHIPIN). The first three steps correspond to the first function of the package, called *CHIPIN_normalize*, described in “Determining genes with constant expression between conditions/samples”, “Building matrix containing ChIP-seq intensity values” and “Performing the normalization” sections. The additional step is executed by the second function of the package, called *plot_expression*, described in “Profiling ChIP-seq intensity around TSS as a function of gene expression level” section.

The output of our package consists of normalized.bigWig files which are directly viewable on the IGV software. The normalized profiles can be used as input into software for calling differential binding, *e*.*g*., HMCan-diff [16].

### Determining genes with constant expression between conditions/samples

The CHIPIN method is based on the assumption that, on average, no difference in true ChIP-seq signal should be observed in the regulatory regions of genes whose expression stays constant across samples/conditions. These genes are determined by the package using gene expression data (RNA-seq or micro-array). For this purpose, we compute the mean and the standard deviation of the Count Per Million (CPM) values for each gene from RNA-seq experiments or unlogged microarray values. To extract an equal percentage of “constant genes” in each range of expression values, we divide all genes into 100 equal-size groups according to the mean values of gene expression. We define the least variable, “constant” genes as genes that show the smallest standard deviation values across samples/conditions (default: 10%) in each expression group (Fig. 1B, “constant genes” are depicted in red). The output of this step is a standard.bed file with gene coordinates.

In addition to the CPM format, the user can provide expression data in the form of Fragments Per Kilobase per Million mapped reads (FPKM) or Transcripts Per Kilobase Million reads (TPM), where read counts are normalized for the gene length; in this case, CHIPIN will transform the FPKM or TPM counts into values proportional to CPM using available transcript annotations (exon lengths).

### Building matrix containing ChIP-seq intensity values

Using.bigWig density files provided by the user and the .bed file containing coordinates of “constant genes”, CHIPIN computes for each sample/condition the density of signal across “constant genes” and their flanking regions (± n Kb, n = 4 by default) (Fig. 1C). For this step, we use the function “*computeMatrix*” provided by the “deepTools” package [3]. The *computeMatrix* function offers the possibility to rescale all genes to the same length (default: 40 kb). The rescaled gene body regions and their additional flanking regions upstream and downstream of the gene body are then segmented into bins (default bin size: 10 bp). Finally, the average signal per bin for each gene is calculated. The output of this step is one matrix per sample/condition with element corresponding to the cumulated density signal per bin and per gene (Fig. 1C).

Of note, CHIPIN does not explicitly take into account Input or IgG control tracks. Instead, we recommend the users to work with densities tracks already normalized for the background signal using one of the available tools. In this study, we used the .bigWig output of the HMCan software [11]. Indeed, peak calling with HMCan allows for the correction of the ChIP-seq signal for copy number and GC-content biases; it reduces the background noise by subtracting the control signal with a constant reflecting the noise ratio of the ChIP sample.

### Performing the normalization

For each sample/condition, the matrices obtained by “deepTools” at the previous step are used to infer normalization parameters. The user can choose between two normalization strategies: quantile and linear normalization, described below. CHIPIN also provides indicators illustrating the success of normalization process: (i) statistics showing the relative difference between average signal curves before and after the normalization procedure; and (ii) visualization of the signal around transcription start sites (TSSs) of “constant genes” before and after the normalization. We further describe two possible normalization strategies the user can choose from.

#### Linear regression with non-zero intercept

The goal of this step is to make density values in all samples/conditions comparable. Here, we choose one of the conditions as a reference (default: sample with the median signal intensity across all conditions). We separately calculate regression coefficients for each sample using the reference density profile and then normalize density profiles using these coefficients. In more detail, for each sample and the reference we first calculate the average signal per bin from the density matrices computed in “Building matrix containing ChIP-seq intensity values” section (Fig. 1C). Then, we perform linear regression with non-zero intercept on these average intensity values: $\overline{S}_{Current\,Sample}$ versus the reference sample $\overline{S}_{Reference\,Sample}$. For each sample, a linear regression with non-zero intercept provides $\alpha$ and $\beta$ that minimize the sum of square errors *ε* in:

$$\overline{S}_{Current\,Sample} = \alpha *\overline{S}_{Reference\,Sample} + \beta + \varepsilon .$$

Given $\alpha$ and $\beta$ for each sample/condition, the density profile in each condition is then corrected:

$$S_{Current\,Sample\,corrected} = \max \left( {0,\left( {S_{Current\,Sample} - \beta } \right)/\alpha } \right).$$

This transformation is applied on the ChIP-seq signal intensity values available in the original.bigWig files.

#### Quantile normalization

To perform quantile normalization, ChIP-seq signal intensity matrix calculated in “Building matrix containing ChIP-seq intensity values” section is first averaged for genes with similar binding intensity (Additional file 2: Figure S2, default number of gene groups $k$ = 20). The quantile normalization is then performed on averaged intensities of these $k$ gene groups (Fig. 1D) and the discovered normalization function is applied to the original ChIP-seq signal intensity values from .bigWig file. The mathematical transformation to obtain the normalized values from the non-normalized values for each group is learned using functions “smooth.spline” and “predict” from the R package “stats”.

### Profiling ChIP-seq intensity around TSS as a function of gene expression level

The CHIPIN package offers the possibility to profile the average ChIP-seq signal around gene TSSs according to gene expression values. Gene expression data, FPKM values or microarray values of all genes, are separated into three groups using *k*-means clustering: high-, medium- and low-expressed genes. Then the ChIP-seq signal around TSSs of these three groups of genes is visualized using a density profile (see “Qualification of the specificity of the antibody used” section for an example of application).

## Results

We validated CHIPIN in comparison with three other normalization techniques: (i) normalization to the same number of reads, (ii) the LILY method based on the idea of matching the signal in the strongest shared peaks [10], and (iii) one of the most recent normalization methods, ChIPSeqSpikeInFree, based on matching the signal in genomic sliding windows via the calculation of turning points in density distributions [6]. We included the normalization to the same number of reads into the comparison because it has been so far the most widely used normalization technique for ChIP-seq signals. And indeed, when applied to conditions characterized within the same technical replicate, this normalization strategy provides stable and trustable results. The two other methods, although not covering the whole spectrum of normalization approaches, are representative of the two hypotheses behind normalization strategies: the one based on the strongest and the other – on overall signal.

To perform the comparison, we used ChIP-seq densities (.bigWig files) obtained with the HMCan software [11]. In our comparison of the methods, we addressed two questions: (i) Can the CHIPIN normalization strategy efficiently match signal densities across conditions and across replicates? and (ii) Does the CHIPIN normalization preserve biological differences between protein binding density signals?

### CHIPIN efficiently matches average density profiles for regions surrounding the TSS of “constant genes”

We assessed the performance of CHIPIN to match ChIP-seq density profiles in genomic regions corresponding to genes with low transcriptional variation across conditions. This analysis was performed on two data sets: five human adrenocortical carcinoma samples profiled for H3K27ac (unpublished data) and the erythroleukemic murine cell line, with doxycycline inducible shRNA against Spi1, further called shSpi1-A2B profiled for H3K27ac in two conditions: (1) with Spi1-overexpressed: Spi1++, and (2) Spi1-repressed: Spi1− [17].

#### Results for human adrenocortical carcinoma samples

**Fig. 2:**
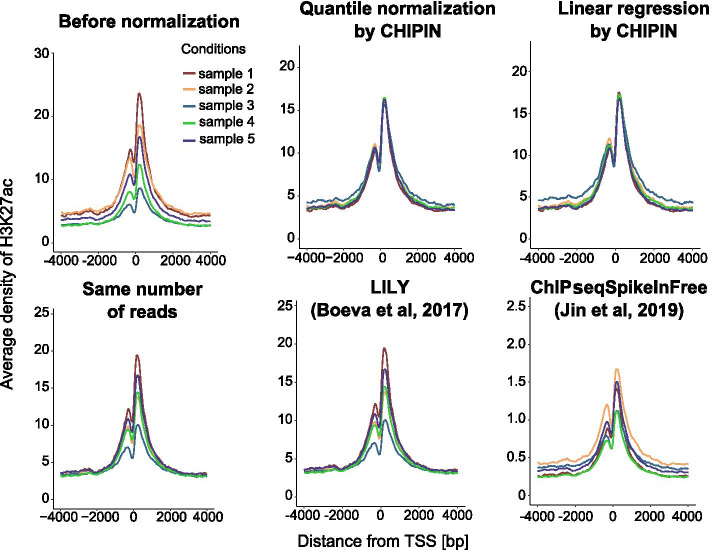
Comparison of normalization efficiency on five H3K27ac data sets of adrenocortical carcinoma. Average density signal is shown for 8 kb regions surrounding TSSs of “constant genes”. The first plot corresponds to the average signal before the normalization. Antibody: ab4729 (rabbit polyclonal, Abcam)

First, we applied two CHIPIN normalization methods (linear regression and quantile normalization) and the three other techniques to H3K27ac profiles obtained for five human adrenocortical carcinoma samples. To assess the effect of the normalization procedure, we calculated and compared the average ChIP-seq density values around TSSs of “constant genes” before and after the normalization. In contrast to the three other methods, both linear and quantile normalization by CHIPIN allowed to remove significant differences between the H3K27ac average density values for this group of low-variability genes (Fig. 2).

We quantified the differences between the density curves of the five samples before and after each normalization procedure (Table 1). For that, we divided the region of 8 kb around the TSSs of “constant genes” into 3 zones, Zone 1 (− 4 Kb, − 1 Kb), Zone 2 (− 1 Kb, + 1 Kb), and Zone 3 (+ 1 Kb, + 4 Kb) (Fig. 1E). The discrepancy between each sample/condition was evaluated by the average difference in percentage of area under the density curves (Table 1). In the central region (Zone 2) corresponding to promoter regions and involved in the regulation of gene expression, CHIPIN achieved much better performance than the other approaches. Indeed, initial difference of 34.75% before the normalization was shrunk by CHIPIN to only 1.8% (quantile normalization), while the best public method, LILY, resulted in 18% of difference between the normalized profiles.

**Table 1:** Average percentage difference between average density curves (Fig. 2) in the three zones surrounding TSSs of “constant genes” (Fig. 1E)

	Zone 1 (%)	Zone 2 (%)	Zone 3 (%)
Before normalization	27.5	34.75	28.25
Quantile Normalization by CHIPIN	15.3	**1.8**	7.9
Linear Regression by CHIPIN	21	4.5	19.2
Same number of reads	7.5	23	**6.5**
LILY	**6.5**	18	6.7
ChIPseqSpikeInFree	29	27	29.5

The smallest value for each zone is shown in bold

#### Results for murine shSpi1-A2B cell line

**Fig. 3:**
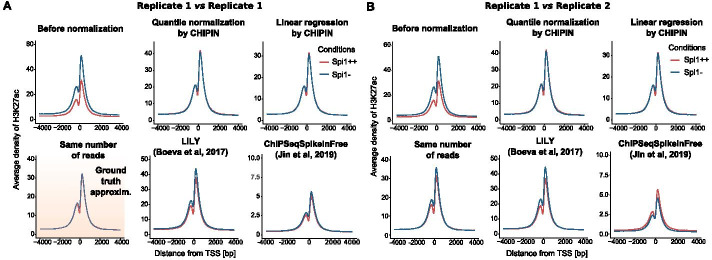
Comparison of normalization efficiency for H3K27ac profiles in the shSpi1-A2B cell line within replicates (**A**) and across replicates (**B**). Average density signal is shown for 8 kb regions surrounding TSSs of “constant genes”. The first plot corresponds to the average signal before the normalization. Antibody: rabbit polyclonal against H3K27ac (ab4729, Abcam); conditions: Spi1++—Spi1 overexpressed, Spi1−—Spi1 repressed by a doxycycline-inducible shRNA

To further validate the performance of CHIPIN, we analyzed the second data set, the shSpi1-A2B cell line profiled for H3K27ac in two conditions: (i) Spi1-overexpressed: Spi1++, and (ii) Spi1-repressed: Spi1−. For each condition, we had two available technical replicates: replicate 1 and 2. To show the importance of correction for the batch effect, we further used replicate 1 for the Spi1++ condition and replicate 2 for the Spi1− condition for the validation of the CHIPIN method. At the same time, normalization to the same number of reads within Replicate 1 provided us with the ground truth (Fig. 3A).

We applied CHIPIN (linear and quantile normalization) and the three other normalization techniques to H3K27ac densities calculated for Spi1++ and Spi1− conditions (Fig. 3B, Table 2). CHIPIN resulted in almost perfect match of the two average density curves for “constant genes” using both quantile normalization and linear regression (0.06% and 0.1% of difference after the normalization *vs* 38% of difference before the normalization for the central Zone 2). Application of all other methods also allowed shrinking the difference in ChIP-seq signals between the two conditions (from 38% to 7.6%, 11.9% and 17.2% for the normalization using same number of reads, ChIPSeqSpikeInFree and LILY, respectively), without however reaching the performance of CHIPIN (Fig. 3B, Table 2).

**Table 2:** Percentage difference between H3K27ac density curves (Fig. 3B) in the three zones surrounding TSSs of “constant genes” (Fig. 1E)

	Zone 1 (%)	Zone 2 (%)	Zone 3 (%)
Before normalization	32	38	30
Quantile Normalization by CHIPIN	4.6	**0.06**	8.6
Linear Regression by CHIPIN	**1.5**	0.1	5.3
Same number of reads	2.8	7.6	2.3
LILY	6.8	17.2	**1.9**
ChIPSeqSpikeInFree	15.6	11.9	18.2

The smallest value for each zone is shown in bold

### CHIPIN preserves biological differences in ChIP-seq density signals between conditions

**Fig. 4:**
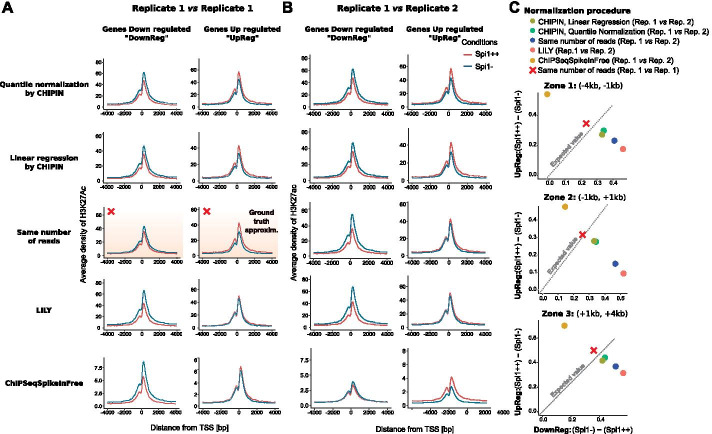
Comparison of normalization efficiency by CHIPIN and the three other methods in genomic regions corresponding to differentially expressed genes in the shSpi1-A2B cells (across replicates). **A** H3K27ac density profiles around TSSs of genes down- and up-regulated by Spi1 in two conditions: “Spi1++”—Spi1 overexpressed, “Spi1−”—Spi1 repressed. **B** Differences in H3K27ac signal in zones 1–3 (Fig. 1E) in gene promoters for genes up- and down-regulated by Spi1; axes show differences between density values in conditions Spi1++ and Spi1−. Correct normalization procedures would result in observations laying close to the diagonal ($y = x$, grey dotted line)

As the CHIPIN normalization procedure uses exclusively genomic regions surrounding “constant genes”, we assessed the method efficiency for maintaining biological differences in ChIP-seq signal for genes that are differentially expressed across the conditions. We extracted genes differentially expressed between the Spi1++ and the Spi1− conditions in the shSpi1-A2B mouse cell line (FC > 1.5, FDR-adjusted t-test p-value < 0.05) and then analyzed density profiles of the activator histone mark H3K27ac in the vicinity of TSS of genes up- and down-regulated in the Spi1++ condition after applying the normalization procedures (Fig. 4).

Given the strong linear correlation between gene transcriptional levels and the H3K27ac promoter signal (Pearson R = 0.83, Additional file 3: Figure S3), we could estimate the expected shift between H3K27ac densities around gene TSSs in the Spi1++ and Spi1− conditions for up- and down-regulated genes: predicted average shifts for down- and up-regulated genes were -22.7 and 19.2, respectively, which corresponded to the ratio of 1.18 between the absolute shift values (Fig. 4C). Indeed, the within replicate normalization for the same number of reads resulted in comparable shift ratio of the signals (Figs. 4C and S2), corroborating our modelling strategy.

When normalizing across conditions, out of the four tested methods, CHIPIN (using both linear and quantile normalization) provided the results closest to the expected (Fig. 4B, C). Application of LILY and the normalization to the same number of reads resulted in differences between H3K27ac density curves in the Spi1++ and Spi1− conditions being much stronger for genes down-regulated by Spi1 than for the up-regulated genes (Fig. 4A). Inversely, the normalization by ChIPSeqSpikeInFree resulted in a much higher differences in the H3K27ac densities for the up-regulated than for the down-regulated genes. Thus, we concluded that CHIPIN outperformed on this biological example the three other methods.

### Qualification of the specificity of the antibody used

The quality of a ChIP-seq experiment relies a lot on the efficiency and the specificity of the antibody used. Indeed, using unspecific antibodies may lead to erroneous biological conclusions. Thus, profiling the average ChIP-seq signal around gene TSSs according to gene expression values is a good way to verify that known biological patterns are respected [18]. One can use *a priory* known information about the effects of binding of the protein of interest on gene expression: highly expressed genes tend to have stronger signal of “active” histone marks (*e*.*g*., H3K27ac and H3K4me3) and transcription factor binding around in their promoters, while silent genes or genes with low expression tend to have stronger signal of “repressor” histone marks (*e*.*g*., H3K27me3 and H3K9me3) in the vicinity of their TSS [19].

**Fig. 5:**
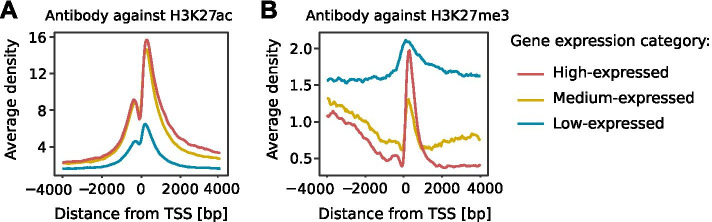
Graphs generated by CHIPIN allowing evaluating specificity of two antibodies used: **A** antibody ab4729 (Abcam) against H3K27ac in an adrenocortical carcinoma sample (unpublished), and **B** antibody ACM39155 (Active Motif) against H3K27me3 in the shSpi1-A2B cells (unpublished). For ab4729, as expected, highly expressed genes show a higher level of H3K27ac than genes of medium or low expression; while for ACM39155, highly expressed genes show an increase of intensities for H3K27me3 downstream TSS (red). This suggests a potential non-specific binding of this antibody targeting H3K27 methylation to acetylated lysines, also documented by Rothbart et al. [20]

Here, we provide an example how the *plot_expression* function of the CHIPIN package allowed detection on unspecific binding of an antibody (Fig. 5).

We applied CHIPIN on two ChIP-seq experiments: H3K27ac in adrenocortical carcinoma samples (antibody ab4729, Abcam) and H3K27me3 in the shSpi1-A2B cells (antibody ACM39155, Active Motif). The histone modification ChIP-seq data were coupled with RNA-seq experiments measuring gene expression in the corresponding samples. CHIPIN generated profiles of average density distributions around TSSs for three groups of genes: low, medium and highly expressed genes (Fig. 5).

As expected, for the adrenocortical carcinoma samples, the density around the gene TSS of the profiled H3K27ac mark positively correlated with gene expression confirming the specificity and high quality of the antibody used (Fig. 5A). Surprisingly however, in the shSpi1-A2B cells the highly expressed genes (Fig. 5B, red) showed a very high average density signal of H3K27me3 in gene immediate downstream region (up to 1 kb downstream TSS). This increase was also seen at some extent for the medium- and low-expressed genes and was likely to be linked to a non-specific binding of the antibody ACM39155 targeting H3K27 methylation we used for this experiment. Indeed, a recent study of antibody specificities showed that, in addition to the targeted repressive mark H3K27me3, the Active Motif antibody 39155 could also bind to several activator marks such as acetylated lysines [20]. This example demonstrated the utility of CHIPIN in assessing antibody specificities and in evaluating quality of a ChIP-seq data set.

## Discussion

We provide a new R package, CHIPIN, for inter-sample or inter-condition normalization of ChIP-seq density signals, which is a mandatory step to enable comparison of transcription factor or histone mark binding intensities across biological conditions. We provide two choices of normalization methods: linear regression with non-zero intercept and quantile normalization. Both methods are more efficient than other published normalization approaches we tested (Figs. 2, 3, 4).

CHIPIN uses as input .bigWig files that are generated by the majority of peak callers. However, in our validation study we gave preference to the HMCan software [11]. Indeed, HMCan is a peak-caller that corrects for the GC-content and copy number biases and removes the background signal if an Input experiment is provided. The use of HMCan is strongly recommended for the comparison of cancer samples with differences in the copy number aberration profiles.

Our method was initially developed for ChIP-seq data and it can be used without any limitation to other density profiles such as those constructed for ATAC-seq or DNase hypersensitivity data. Recently, a study by Reske et al. [21] demonstrated that the choice of the normalization method for the comparison of chromatin accessibility in different regions, from ATAC-seq experiments, was of crucial importance in helping avoid false biological interpretations. As it has been reported that promoter accessibility positively correlates with gene expression [22], using genes whose expression is constant across conditions/samples, as implemented in CHIPIN, represents an appropriate solution for normalizing ATAC-seq data.

Of note, our method should not be used when the targeted protein is overexpressed or silenced in one of the samples/conditions. Instead, spike-in protocols should be employed, followed by a normalization with an appropriate method accounting for spike-in information, *e*.*g*., HMCan-diff [16], to determine differentially enriched regions.

Here, we used two data sets to compare CHIPIN with other available normalization methods. For the second data set, profiling H3K27ac in the shSpi1-A2B cell line in two conditions, two replicates were available for each condition. For the validation of our method, we chose replicate 1 for the first condition and replicate 2 for the second condition to have a stronger batch effect. In this comparison, CHIPIN outperformed the other methods (Figs. 3, 4; Tables 1, 2). However, we also assessed the performance of CHIPIN in the setting where we were normalizing ChIP-seq density profiles obtained within the same replicate/batch (Fig. 3A, 4A, Additional file 4: Figure S4 and Additional file 5: Table S1). Importantly, in this case as well, CHIPIN outperformed the other normalization methods. It could be however seen that the normalization to the same number of reads also provided almost perfect results (Additional file 5: Table S1, Additional file 4: Figure S4). This analysis demonstrated the importance of inter-sample normalization when comparing different replicates, while the normalization to the same number of reads could be applied on experiments performed within the same batch.

## Conclusions

Here, we present a novel method for the inter-condition normalization of ChIP-seq data when spike-in data are not available. The method is based on the assumption that, on average, protein binding in regulatory regions of genes stably expressed or silent across the conditions is also constant across the conditions. We provide a choice of normalization parameters and options. In particular, the user can choose between a linear normalization with a non-zero intercept and quantile normalization. The method is implemented in a user-friendly R package available on GitHub: https://github.com/BoevaLab/CHIPIN. Several examples are presented in the GitHub vignette and test data are provided.

## Availability and requirements

Project name: CHIPIN

Project home page: https://github.com/BoevaLab/CHIPIN

Operating system(s): Linux

Programming language: R

Other requirements: deepTools

License: GNU GPL

Any restrictions to use by non-academics: no restriction

## Supplementary Information

**Additional file 1: Figure S1.** ChIP-seq density profiles for selected transcription factors in the K562 cell line as a function of gene expression. ENCODE data sets used: ENCSR492FKD (ZNF257), ENCSR271RRH (ZNF253), ENCSR121SPB (KLF10), ENCSR844JVU (TCFL5), ENCSR946BXO (BRCA1), ENCSR153HNT (STAG1), ENCSR549PVK (PATZ1), ENCSR017EJY (DLX4). Gene expression was assessed an average across two replicates: ENCFF928NYA and ENCFF003XKT [23].

**Additional file 2: Figure S2**. Gene group definition of the quantile normalization. The quantile normalization is performed on the concatenated binding intensities averaged over *k* groups of genes (default *k* = 20); gene groups are defined based on the strength of the overall binding signal (averaged across all samples), so that each gene group corresponds to the specific ChIP-seq signal intensity: from the lowest (group 1) to the highest (group *k*). To obtain gene groups, we concatenate matrices with ChIP-seq density values (built by deepTools) over all samples (**A**, two samples are shown), compute the average values of signal intensity across bins and samples for each gene (**B**), sort genes according to the overall average intensity values (**C**), and then build *k* gene groups by splitting the whole set of genes (**D**) so that each gene group represents a certain range of ChIP-seq intensity signal.

**Additional file 3: Figure S3.** Linear regression fit between gene expression in the Spi1++ condition and H3K27ac densities. Gene expression was evaluated in FPKM, and ChIP-seq H3K27ac maximal density corresponds to the maximal density in the 2 Kb window surrounding gene TSS averaged between Replicate 1 and 2. The ChIP-seq signal was normalized for the CG-content, copy number bias and background noise by HMCan [11].

**Additional file 4: Figure S4.** Comparison of normalization efficiency by CHIPIN and the three other methods in genomic regions corresponding to differentially expressed genes in the shSpi1-A2B cells (replicate 1 is used for both conditions). Antibody: ab4729 (Abcam) against H3K27ac; **A** H3K27ac density profiles around TSSs of genes down- and up-regulated by Spi1 in two conditions: “Spi1++ ”—Spi1 overexpressed, “Spi1−”—Spi1 repressed. **B** Differences in H3K27ac signal in zones 1–3 (Fig. 1E) in gene promoters for genes up- and down-regulated by Spi1; axes show differences between density values in conditions Spi1++ and Spi1−. Correct normalization procedures would result in observations laying close to the diagonal ($y = x$, grey dotted line).

**Additional file 5: Table S1.** Percentage difference between H3K27ac density curves (Fig. 3A) in the three zones surrounding TSSs of “constant genes” (Fig. 1E).

## Data Availability

The code of CHIPIN, examples and readme are available at https://github.com/BoevaLab/CHIPIN. Unpublished ChIP-seq data sets used in this study are available upon the request.

## Abbreviations

ChIP-seq: Chromatin immunoprecipitation followed by sequencing
CPM: Count Per Million
FPKM: Fragments Per Kilobase per Million mapped reads
TPM: Transcripts Per Kilobase Million reads
TSS: Transcription Start Site
