# Supplementary figures and images for: CHIPIN: ChIP-seq inter-sample normalization based on signal invariance across transcriptionally constant genes
Source: BMC Bioinformatics. 2021 Aug 17;22:407. doi: 10.1186/s12859-021-04320-3 (PMC8371782; doi:10.1186/s12859-021-04320-3)

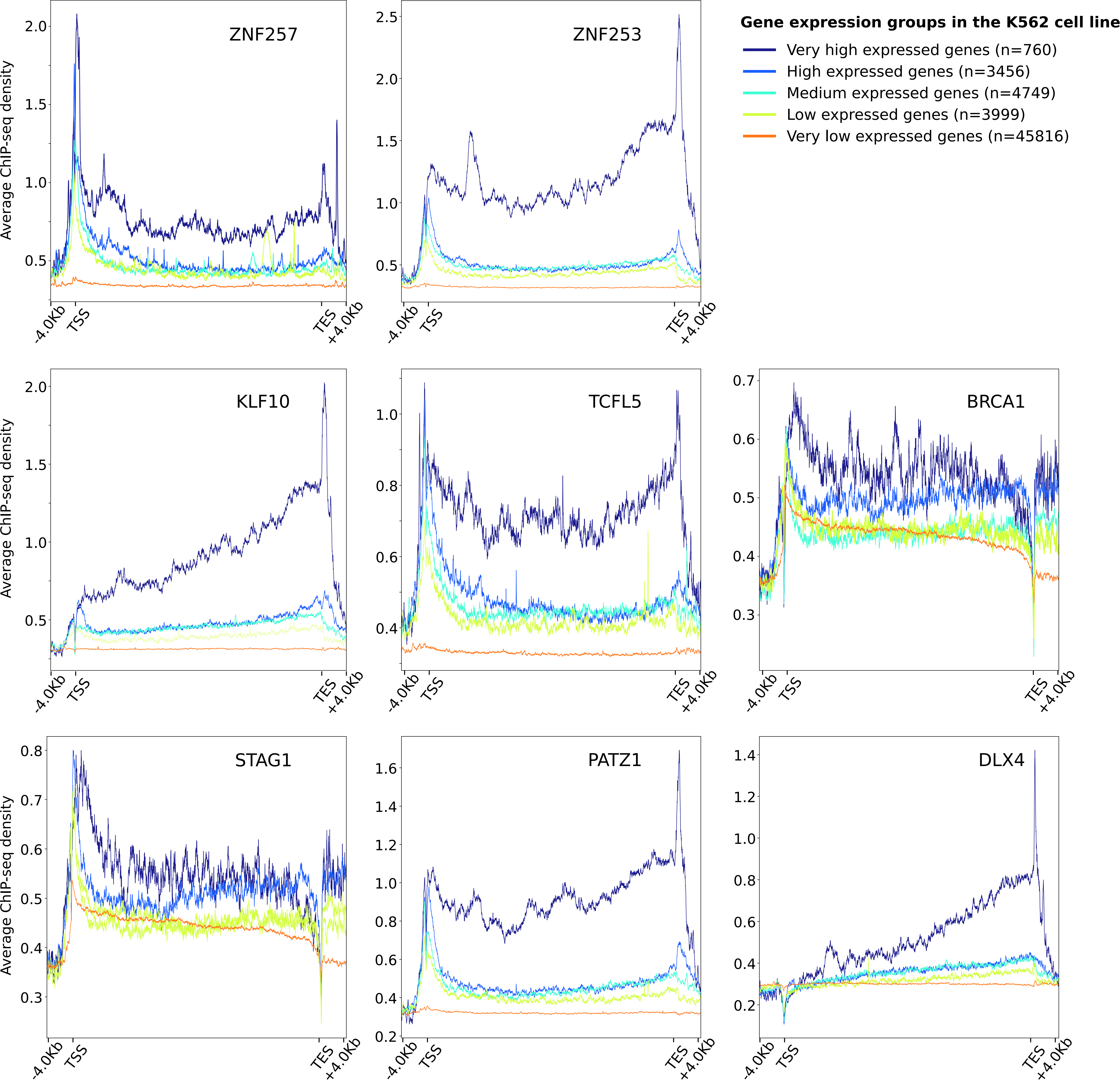

Supplement: Supplementary file 1 — Additional file 1: Figure S1. ChIP-seq density profiles for selected transcription factors in the K562 cell line as a function of gene expression. ENCODE data sets used: ENCSR492FKD (ZNF257), ENCSR271RRH (ZNF253), ENCSR121SPB (KLF10), ENCSR844JVU (TCFL5), ENCSR946BXO (BRCA1), ENCSR153HNT (STAG1), ENCSR549PVK (PATZ1), ENCSR017EJY (DLX4). Gene expression was assessed an average across two replicates: ENCFF928NYA and ENCFF003XKT [23]. [file 12859_2021_4320_MOESM1_ESM.pdf]

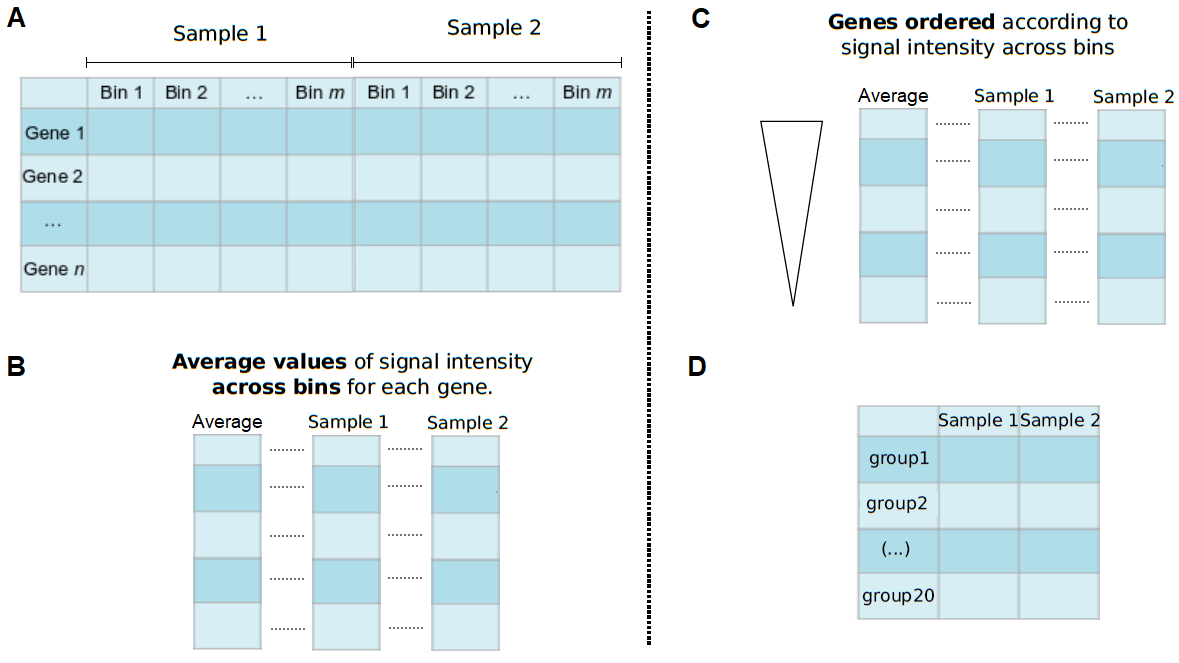

Supplement: Supplementary file 2 — Additional file 2: Figure S2. Gene group definition of the quantile normalization. The quantile normalization is performed on the concatenated binding intensities averaged over k groups of genes (default k = 20); gene groups are defined based on the strength of the overall binding signal (averaged across all samples), so that each gene group corresponds to the specific ChIP-seq signal intensity: from the lowest (group 1) to the highest (group k). To obtain gene groups, we concatenate matrices with ChIP-seq density values (built by deepTools) over all samples (A, two samples are shown), compute the average values of signal intensity across bins and samples for each gene (B), sort genes according to the overall average intensity values (C), and then build k gene groups by splitting the whole set of genes (D) so that each gene group represents a certain range of ChIP-seq intensity signal. [file 12859_2021_4320_MOESM2_ESM.png]

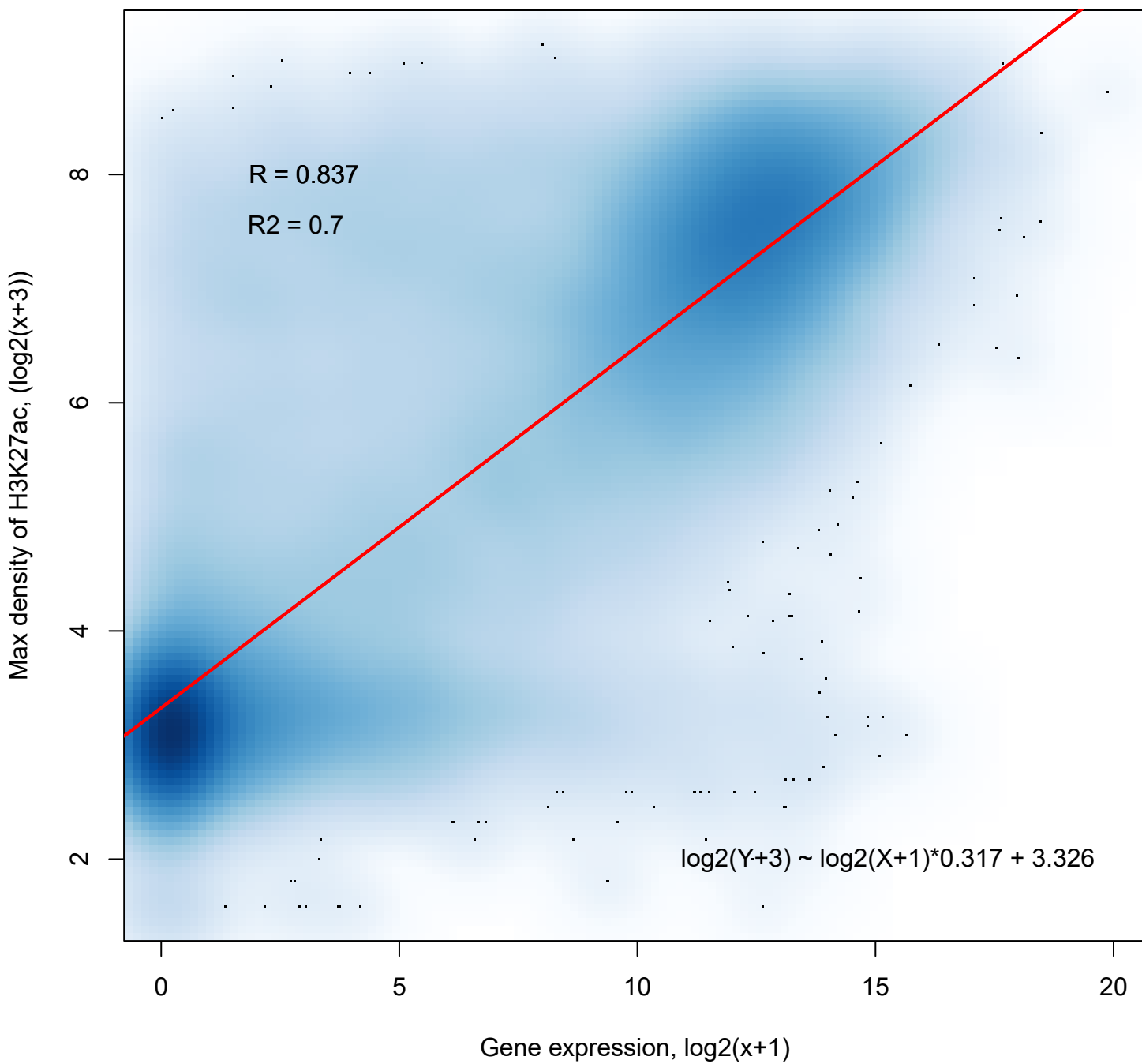

Supplement: Supplementary file 3 — Additional file 3: Figure S3. Linear regression fit between gene expression in the Spi1++ condition and H3K27ac densities. Gene expression was evaluated in FPKM, and ChIP-seq H3K27ac maximal density corresponds to the maximal density in the 2 Kb window surrounding gene TSS averaged between Replicate 1 and 2. The ChIP-seq signal was normalized for the CG-content, copy number bias and background noise by HMCan [11]. [file 12859_2021_4320_MOESM3_ESM.pdf]
