# Supplementary material for: CHIPIN: ChIP-seq inter-sample normalization based on signal invariance across transcriptionally constant genes
Source: BMC Bioinformatics. 2021 Aug 17;22:407. doi: 10.1186/s12859-021-04320-3 (PMC8371782; doi:10.1186/s12859-021-04320-3)
Supplement: Supplementary file 4 — Additional file 4: Figure S4. Comparison of normalization efficiency by CHIPIN and the three other methods in genomic regions corresponding to differentially expressed genes in the shSpi1-A2B cells (replicate 1 is used for both conditions). Antibody: ab4729 (Abcam) against H3K27ac; A H3K27ac density profiles around TSSs of genes down- and up-regulated by Spi1 in two conditions: “Spi1++ ”—Spi1 overexpressed, “Spi1−”—Spi1 repressed. B Differences in H3K27ac signal in zones 1–3 (Fig. 1E) in gene promoters for genes up- and down-regulated by Spi1; axes show differences between density values in conditions Spi1++ and Spi1−. Correct normalization procedures would result in observations laying close to the diagonal ($y = x$y=x, grey dotted line). [file 12859_2021_4320_MOESM4_ESM.pdf]

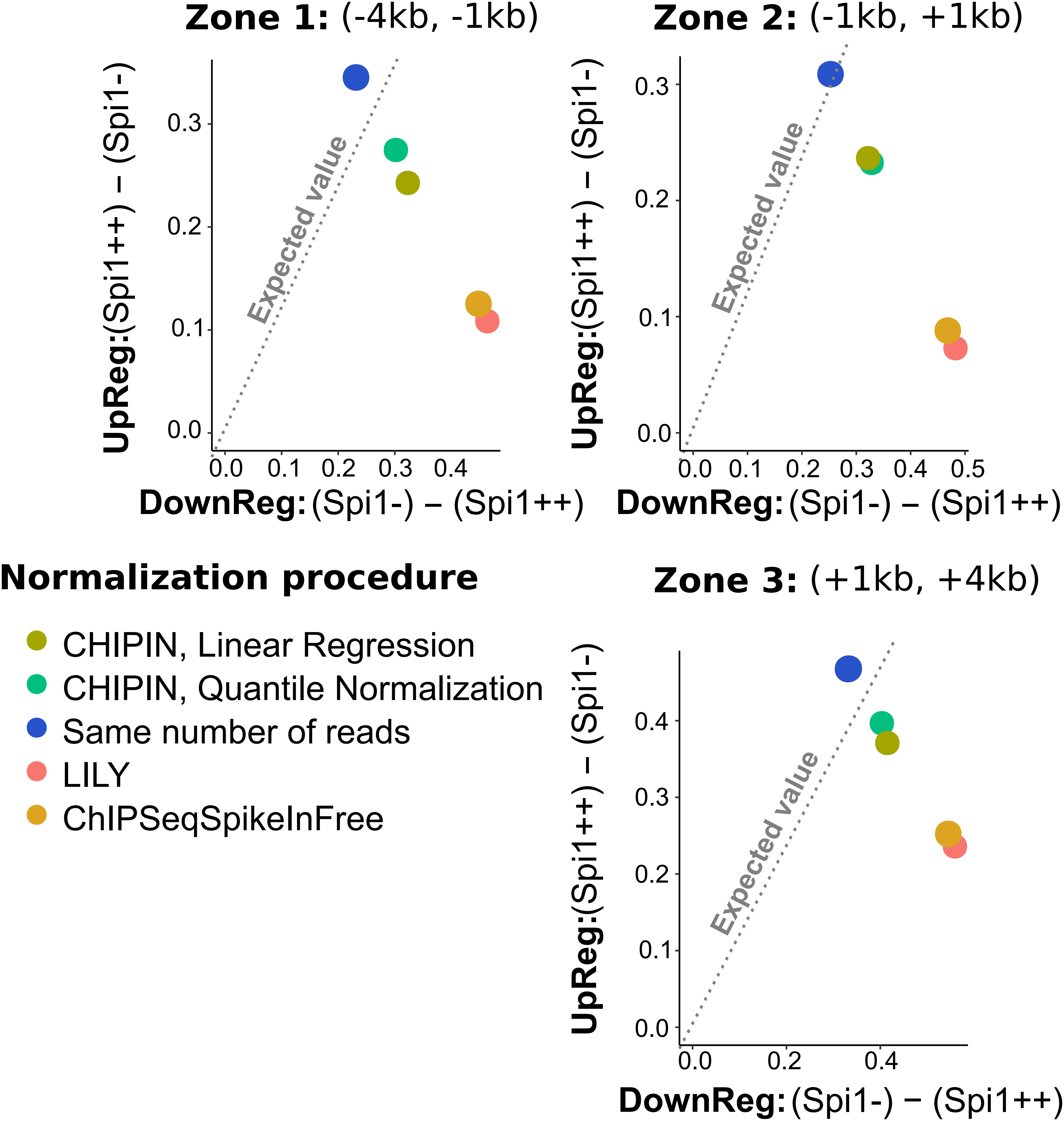
Zone 1: (-4kb, -1kb)
Expected value
UpReg:(Spi1++) − (Spi1-)
0.0
0.1
0.2
0.3
0.0 0.1 0.2 0.3 0.4
DownReg: (Spi1-) − (Spi1++)
Zone 2: (-1kb, +1kb)
Expected value
UpReg:(Spi1++) − (Spi1-)
0.0
0.1
0.2
0.3
0.0 0.1 0.2 0.3 0.4 0.5
DownReg: (Spi1-) − (Spi1++)
Normalization procedure
CHIPIN, Linear Regression
CHIPIN, Quantile Normalization
Same number of reads
LILY
ChIPSeqSpikeInFree
Zone 3: (+1kb, +4kb)
Expected value
UpReg:(Spi1++) − (Spi1-)
0.0
0.1
0.2
0.3
0.4
0.0 0.2 0.4
DownReg: (Spi1-) − (Spi1++)
