# Supplementary material for: CHIPIN: ChIP-seq inter-sample normalization based on signal invariance across transcriptionally constant genes
Source: BMC Bioinformatics. 2021 Aug 17;22:407. doi: 10.1186/s12859-021-04320-3 (PMC8371782; doi:10.1186/s12859-021-04320-3)
Supplement: Supplementary file 5 — Additional file 5: Table S1. Percentage difference between H3K27ac density curves (Fig. 3A) in the three zones surrounding TSSs of “constant genes” (Fig. 1E). [file 12859_2021_4320_MOESM5_ESM.docx]

|  | **Zone 1** | **Zone 2** | **Zone 3** |
| --- | --- | --- | --- |
| **Before normalization** | 37% | 40% | 35% |
| **Quantile Normalization by CHIPIN** | 3.6% | 0.7% | 6.5% |
| **Linear Regression by CHIPIN** | **0.09%** | **0.6%** | **2.6%** |
| **Same number of reads** | 0.7**%** | 3.6% | 3.4**%** |
| **LILY** | 12.2% | 15.6% | 9.6% |
| **ChIPSeqSpikeInFree** | 15.7% | 11.9% | 18.2% |

**Table S1**. Percentage difference between H3K27ac density curves (Figure 3A) in the three zones surrounding TSSs of “constant genes” (Figure 1E).
